# Economic Impact of an Advanced Illness Consultation Program within a Medicare Advantage Plan Population

**DOI:** 10.1089/jpm.2015.0423

**Published:** 2016-06-01

**Authors:** Vincent Colaberdino, Colleen Marshall, Paul DuBose, Mitchell Daitz

**Affiliations:** ^1^ Vital Decisions, Cherry Hill, New Jersey.; ^2^ Principled Strategies, Encinitas, California.

## Abstract

***Background:*** In the United States the quality and cost associated with medical treatment for individuals experiencing an advanced illness is highly variable and is often misaligned with the patient's and family's quality of life values and priorities. Many of the obstacles that stand in the way of aligning the care that an individual receives with their priorities are well understood in the context of behavioral science. Through employing behavioral based approaches to improve the quality of communication and shared decision making processes among patients, providers and families it is possible to enhance the efficiency of delivering care which is also more highly aligned with the individual's preferences.

***Objectives:*** The study objectives were to measure the economic impact of a proprietary advanced illness behavioral consultation program, the Vital Decisions Living Well Consultation Program (LWCP), on the cost of care delivered during the last six and three months of life for enrolled members in a Medicare Advantage plan.

***Study design:*** Retrospective matched case control analysis examined medical, pharmaceutical, and capitation expenses after an offset by premium revenue.

***Methods:*** The treatment population consisted of participating members of the LWCP who died between October 1, 2011 and March 31, 2013 (*N* = 1755). The control population consisted of plan members who died between July 1, 2008 and June 1, 2011, prior to the introduction of the LWCP (*N* = 5560). Criteria used to match treatment subjects with one or more control subjects were diagnosis, age at death, and health care costs incurred prior to the time under examination. A paired t-test evaluated the significance of differences between the matched treatment and control members.

***Results:*** The mean cost reduction during the last six months of life for treatment members compared to matched control group members was $13,956 (*p* < 0.001) during the last six months of life and $9,285 (*p* < 0.001) during the last three months of life.

***Conclusion:*** Members with advanced illness who participated in LWCP had significantly lower end-of-life (EOL) spending compared to matched members who did not participate in the program.

## Introduction

U. S. health care costs are increasing overall, especially costs for aging patients.^1^ Morhaim (2013)^2^ states, “For most Americans, it is estimated that 25% or more of all the health care dollars spent in their entire lives are spent in the last months of life.” Research has shown that some end-of-life (EOL) costs may be the result of futile care or aggressive care that is misaligned with patient care preferences.^3–4^

It is widely noted that effective communication and decision-making processes among participants in an advanced illness care delivery system can improve quality-of-life outcomes.^5–10^ Simultaneously, when communication processes are aimed at aligning an individual's quality-of-life preferences with the care delivered by the system, an improvement in economic efficiency of care is realized.^11–12^

Nonetheless, initiating and conducting such discussions is difficult and often does not occur.^13–16^ Furthermore, evidence suggests that the traditional approach to initiating this discussion, the process of completing an advance care directive document is not sufficient.^17,18^ Such historical shortcomings have led to increased focus on broadening the approach of advance care planning beyond a written advance directive. Improving communication and shared decision making among clinicians, patients, and family members, including recognition of the importance of changing the behaviors of the participants in the process, has been demonstrated to drive success.^19–22^

Vital Decisions is an advance care planning organization that specializes in facilitating the alignment of care with an individual's values and priorities through their Living Well Consultation Program (LWCP). The objective of Vital Decisions' LWCP is to improve an individual's ability to implement high-quality communications and shared decision making processes with physicians and family to align health care interventions with patient quality-of-life preferences and values. The LWCP consists of an advanced-degree expert who telephonically interacts with the patient, and in many cases with their family, to deliver a proprietary program to improve the individual's communication and shared decision making processes. The program, conducted over multiple sessions, identifies obstacles such as fear, denial, lack of self-confidence, and family dynamics—that may be inhibiting an individual's ability to communicate preferences and values to their family and/or physicians. The intervention develops the intrinsic motivation to overcome these barriers, and enables the communication of an individual's values and preferences so they may serve as the basis for current and future care decision making. The goal is to focus the process on five primary target behaviors of the patient: being medically informed of one's current medical situation and future prognosis, evaluating how the priorities and preferences of the individual align with the options available, communicating those priorities and making care decisions with providers and family stakeholders that align with these values, and repeating the process as shift points occur in the patient's situation.

The hypothesis underpinning the LWCP methodology, and the hypothesis under examination, is that the LWCP results in overall lower medical expenditures during the last months of life while simultaneously providing the participant with a favorable experience and a high degree of satisfaction. This article reports the results of an analysis evaluating this hypothesis.

## Methods

Medicare Advantage plan members who were identified by Vital Decisions as experiencing an advanced illness and who agreed to engage in the program were entered into the potential intervention group. Potential intervention group members also were subject to the requirement of being engaged in the LWCP for a minimum of 10 days in the three-month analysis and a minimum of 120 days in the six-month analysis, and died between October 1, 2011 and March 31, 2013. Members of the potential control group were Medicare Advantage members who died between July 1, 2008 and June 30, 2011. During this time, the LWCP was not available to Medicare Advantage plan members. By using an historic control group, some self-selection bias is avoided. Table 1 describes the potential intervention and potential control groups for the analysis.

**Table 1.** Populations Available for Finding Matched Intervention and Control Sets

*Analysis group*	*Count*	*Mean age at death*	*Percentage female*
Potential intervention	N = 1755	81.1 years	51.2%
Potential control	N = 5560	80.7 years	51.7%

Monthly patient medical costs included medical, pharmacy, and capitation costs incurred by the Medicare Advantage on behalf of the member. These costs were offset by the monthly amount that the plan received as a premium payment for the individual. Medical costs for both the intervention and control groups used amounts based on completed, final claims. Since control group death dates were earlier than intervention death dates, a medical inflation trend factor was used to adjust control group decedents' costs.

A boxplot analysis was used to identify outlier boundaries for the six-month analysis and the three-month analysis. Decedents in either the top or bottom 1.5% of all members for total spending in six months prior to the final six months before death were removed from the six-month analysis. Members in the bottom 1.5% of all members for total spending in the final six months were removed from the six-month analysis. Total spending for the final six months for all intervention and control members was capped at $200,000.

Decedents in the top or bottom 1.5% of all members for total spending in nine months prior to the final three months before death were removed from the three-month analysis. Members who fell in the bottom 1.5% of all members for total spending in the final three months were removed from the three-month analysis. Total spending for the final three months for all intervention and control members was capped at $200,000 minus the total spending in the prior three months, since a $200,000 six-month spending cap was in place for all members.

The use of a historical control group provided several advantages over controls from identified, but non-LWCP-engaged decedents during the same period. Utilizing identified-only decedents as controls could result in self-selection bias, and the historical control group provided a much larger population. Adjusting for unit cost differences over time was a drawback, but the available cost-trend information was considered sufficient for accurate cost comparisons.

The selected analysis methodology was matched controls. Stuart^23^ provides an overview of matching control methodology, which attempts to “replicate a randomized experiment as close as possible by obtaining treated and control groups with similar distributions…thereby reducing bias due to covariates.”

The three matching parameters for this study were (1) primary death diagnosis (exact match required), (2) member age (difference of less than eight years required), and (3) medical costs prior to the final months of life prior to the analysis period. The medical cost difference between an intervention decedent and a matching control decedent for the six-month analysis was required to be less than $5,000 for the six months prior to the final six months before death or in the case of the three month analysis, less than $5,000 for the nine months prior to the final three months before death.

For each intervened decedent, a search of the control population found all possible decedents who matched the aforementioned criteria. There were three different outcomes for each intervention decedent: (1) no matches, (2) one match, or (3) more than one match. If there were no matches, the intervention decedent was removed from the study. If there were multiple matched controls, average costs were computed for the set of matched controls. Using the average of multiple control matches decreases the variance of the matched control value and thus improves statistical power.^24^

Analysis methodology robustness was evaluated by computing cost reductions for a range of matching criteria for the three-month analysis. Evaluations were conducted for age differences of 4, 8, and 10 years and analysis period cost differences of $2,500, $5,000, and $10,000. Smaller differences in age and medical costs lowered the number of matches by 13% and the estimated decedent savings by 11%. Larger differences in age and medical costs increased the number of matches by 3% and the estimated decedent savings by 4%. These computations suggested that a reasonable range of values for the selected matching criteria would produce a similar analysis result.

## Results

Medical costs incurred by LWCP-engaged decedents were significantly lower in the final six and final three months of life than otherwise similar control decedents. The distribution of the matched differences of medical costs was unimodal and approximately symmetric. Since individual differences were thought to be independent and share the same distribution, the central limit theorem suggests that the mean of the sample of cost differences will approximate a normal distribution. Hence, a paired t-test was appropriate to evaluate the significance of the mean of the cost savings.

Over the last six months of life, 228 intervention members were matched with 3588 controls, and had average savings of $13,956 (*p* = 0.001), representing approximately 35% savings. Over the last three months of life, 386 intervention members were matched with 3755 matched controls, and had average savings of $9,285 (*p* < 0.001), representing approximately 30% savings. After factoring for the costs associated with implementing and operating the program, this represents a program return on investment (ROI) in excess of 6:1 per engaged decedent.

The LWCP has also achieved a high satisfaction rate with members who engaged in the program. A satisfaction survey was sent to engaged members after starting the program and to the family/surrogate after the member's death. This goal of high member and family satisfaction is achieved by fostering communication of the individual's quality-of-life preferences, which often go undiscussed with family and providers. By having these conversations, there is greater likelihood that the treatment the member receives will align with their priorities. An additional outcome is assistance in reducing unresolved guilt on behalf of the surrogates, as they will have clarity that their advocacy on behalf of the member was what the member truly wanted, less influenced by other biases. The satisfaction survey had a 28% response rate as of June 30, 2013. The five-point Likert^18^ scale for responses was 1 = No, not at all; 3 = Yes; and 5 = Yes, very much. Table 2 summarizes the survey results.

**Table 2.** Results from Survey on LWCP Quality

	*Median*
LWCP helped me to focus on what is most important to me as I deal with my illness.	4
LWCP helped and/or supported me in making decisions regarding my medical care.	4
My LWCP specialist was friendly and courteous.	5
My LWCP specialist was sensitive to my situation.	5
I was satisfied with the service that LWCP provided.	5
I would recommend LWCP to others in similar situations.	5

LWCP, Living Well Consultation Program.

## Discussion

Medical costs by LWCP-engaged decedents were significantly lower in the final three and final six months of life than otherwise similar control decedents. Program cost reduction may result from a decrease in unwanted or futile care.

Several studies have found savings associated with EOL programs. Zhang et al.^5^ established savings from patients who reported EOL discussions. Kelly et al.^12^ found savings dependent on length of hospice enrollment.

While achieving economic benefit, the LWCP program was also well received by members and their family/surrogate. This is in part due to the program goal of aligning treatment with member's preferences. Surveys had a median score of 5 (highest score available) for recommending the program, service satisfaction, sensitivity, and courtesy. Median score of 4 occurred for helping focus on important medical issues and help in making medical decisions. In conclusion, a well designed and executed advanced illness program that improves values-based communication and shared decision-making processes among patients, families, and physicians will likely significantly decrease patient costs during the last six months of life, while simultaneously increasing the quality of life for participants and their families.

As the population with advanced illness continues to grow along with the medical expense to treat them, fostering high-quality communication between the stakeholders may become an increasing component of the treatment plan, given the impact it can have on patient satisfaction as well as containing costs.

### Limitations

Since control group death dates were earlier than intervention death dates, a medical inflation trend factor was used to adjust control group decedents' costs. Adjusting for unit cost differences over time was a drawback, but the available cost-trend information was considered sufficient for accurate cost comparisons.

The analysis does not estimate economic value as a function of time-in-program but focuses on savings for two time durations prior to death. The 228 and 386 engaged members matched to control patients might be fewer than desired, although results had strong statistical significance.

Matched control sets analysis relies on fixed-distance comparisons without a methodology to determine optimal distances. For this reason, a range of distances was considered and results were robust to the choice of distances.
